# Mortality and subsequent fractures of patients with olecranon fractures compared to other upper extremity osteoporotic fractures

**DOI:** 10.1177/17585732221124301

**Published:** 2022-09-01

**Authors:** Ida K Rantalaiho, Inari Laaksonen, Joel Kostensalo, Elina M Ekman, Anssi J Ryösä, Ville O Äärimaa

**Affiliations:** 1 Department of Orthopedics and Traumatology, Turku University Hospital, Turku, Finland; 2 8058 University of Turku, Department of Clinical Medicine, Turku, Finland; 3 Natural Resources, 419837 Natural Resources Institute Finland (Luke), Joensuu, Finland

**Keywords:** Olecranon fracture, osteoporosis, osteoporotic fracture, wrist fracture, proximal humerus fracture

## Abstract

**Background:**

The incidence rate of olecranon fractures is highest in the elderly population. The aim of this study was to determine whether patients with olecranon fractures have similar demographic and risk characteristics compared to patients with osteoporotic upper extremity fractures.

**Methods:**

A retrospective data analysis was performed with diagnoses for olecranon fracture, distal radius fracture and proximal humerus fracture between 2014 and 2016.

**Results:**

A total of 157 olecranon, 1022 distal radius and 451 proximal humerus fractures were identified. The risk of mortality after olecranon and distal radius fractures was comparable but statistically significantly higher after proximal humerus fractures (HR 1.97, 95% CI 1.19–3.27). The risk of subsequent osteoporotic fractures after an olecranon fracture was 10% at 1 year and 14% at 5 years and the risks did not differ statistically after a proximal humerus fracture, 6% and 11% (HR 0.65, 95% CI 0.40–1.06). After a distal radius fracture, the risks were statistically significantly lower: 2% and 5% (HR 0.35, 95% CI 0.22–0.56).

**Discussion:**

Patients with olecranon fractures have essentially similar demographic characteristics compared to patients with distal radius fractures, but the probability for a subsequent fracture is significantly higher and more comparable to patients with proximal humerus fractures.

## Introduction

Low bone mineral density and a tendency to fall due to aging are the best predictive factors for non-vertebral fractures (NVF).^1^ The occurrence of one fracture predicts a second fracture.^1^ One report, found a 2.6 relative risk for a subsequent hip fracture after an olecranon fracture when compared to a female background population.^2^ A study of over 2000 patients found that individuals with any NVF had an 18% absolute risk for a subsequent NVF in the following 5 years, with the risk being highest within the first year.^3^ Proximal humerus fractures are amongst the most frequently studied fractures of upper extremity and known to be an independent risk factor for a subsequent hip fracture for women in the first year after the humerus fracture.^4^

Patients with an NVF are reported to have a 32% absolute risk for mortality in the subsequent 5 years, with the risk of mortality being highest during the first year.^3^ The effect of upper extremity fractures on mortality is less studied, but there is evidence that proximal humerus fractures might be associated with increased mortality.^5–8^ Wrist fractures have not been associated with increased mortality in earlier studies.^5,7–9^ In a population-based study from Finland, proximal forearm fractures requiring inpatient care were associated with increased mortality rates compared to standard population during 6 years follow-up.^7^ Despite of theories of olecranon fractures having osteoporotic features, there are only few studies assessing demographics, mortality and risk of subsequent fracture associated with these fractures.

The aim of this study was to assess the demographic features of patients with olecranon fractures, their mortality, and risk for subsequent fractures compared to other common upper extremity fractures. We hypothesized that patients with olecranon fractures are similar to those with other upper extremity fractures.

## Material and methods

The study protocol was approved, and permission granted by The Hospital District of South-West Finland in 2020 (TO1/019/20). The public health service emergency in the city of Turku is centralized at Turku University Hospital taking care of, for example, all fractures since autumn 2013. Patients aged 18 or older, living in the city of Turku, and being treated for an upper limb fracture were identified retrospectively from the electronic patient record system of Turku University Hospital between a 3-year period from 2014 to 2016 in order to allow a minimum five years follow up on all patients. Patients living outside Turku city area were excluded to ensure good coverage of data.

The specific fractures, proximal ulnar fracture, distal radius fracture and proximal humerus fracture, were identified using diagnosis coding (S52.0, S52.5 or S52.60, S42.2, respectively) according to International Statistical Classification of Diseases and Related Health Problems 10th Revision (ICD-10-CM). After the recognition of the initial fracture the patients were followed through medical charts until February 2022 and data on possible subsequent olecranon, distal radius, proximal humerus or hip fractures (S72.0, S72.1 or S72.2), or time of death was retrieved. Mortality is routinely recorded in the databases from patient records or a civil register. In addition, demographic data, such as sex and age, and possible surgeries and their dates related to the identified fractures were retrieved. NOMESCO Classification of Surgical Procedures Version 1.14 from the Nordic Medico-Statistical Committee was applied with operation codes NCJ62 and NCJ64 for olecranon, NCJ62, NCJ64, NCJ70, and NCJ99 for distal radius, and NBL00, NBJ62, NBJ64, NBJ60, NBJ70, NBJ91, NBB10, NBB15, NBB20 for proximal humerus fractures.

### Statistical analysis

The survival function of patients was estimated for each index fracture separately using a Kaplan-Meier (KM) estimator with censoring done at the beginning of February 2022, when the data was updated for the last time.^10^ The effect of age, sex and treatment method, that is, whether the fracture was operated on or not, was investigated using Cox proportional hazard model.^11^ In further exploratory analyses, the association between subsequent fractures and mortality was also explored. The risk of additional fractures simultaneously or in the 5 years following the index fracture was estimated with a KM estimator, with death being treated as a censoring event. Finally, the risk of mortality and subsequent fractures were considered in parallel with competing risk analysis.^12^ The difference in mortality and the likelihood of subsequent fractures between the index fractures was investigated with a competing risk regression model with mortality and an additional fracture as endpoints with age and sex controlled for. While this model makes it possible to compare outcomes between index fractures in the population as a whole, the underlying assumption of the model is that the effects of age and sex are similar for all fractures. The statistical analyses were carried out using the statistical software R.^13^ The KM-estimators and Cox proportional hazard models were fitted using the R package *survival* and the package *cmprsk.*^14^

## Results

During the 3-year period from January 2014 to December 2016, 157 olecranon fractures, 1022 distal radius fractures and 451 proximal humerus fractures were identified. Of patients with olecranon fracture 62% were women, whereas corresponding numbers for distal radius fractures and proximal humerus fractures were 75% and 70%. Fifty-seven percent of the olecranon fractures were operatively treated, whereas only 15% of the proximal humerus fractures were operated (Table 1).

**Table 1.** Demographics of the included patients.

Fracture	Total *N*	Female (%)	Age (median (IQR))			Operated (*N* (%))
			M	F	ALL	
**Olecranon **	157	62.4	51 (32)	70 (32)	65 (37)	90 (57.3)
**Distal radius **	1022	75.3	54 (35)	68 (24)	65 (26)	253 (24.8)
**Proximal humerus **	451	70.3	61 (25)	70 (20)	68 (23)	67 (14.9)

n: number of; IQR: interquartile range; M: male; F: female.

**Figure 1.**
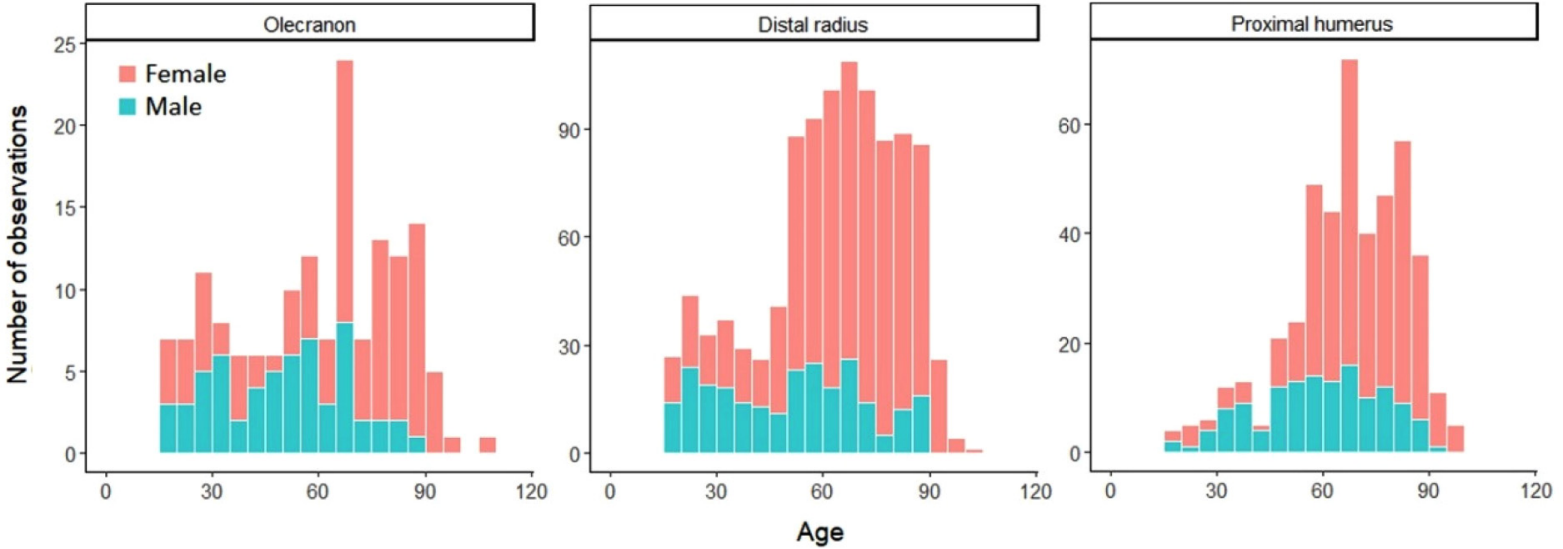
Age distribution for olecranon, distal radius, and proximal humeral fractures.

The median age at the time of the fracture was 65 years for patients with an olecranon and a distal radius fracture (IQR 37 and 26, respectively) and 68 (IQR 23) years for patients with a proximal humerus fracture. The distribution of number of fractures in different age groups is presented in Figure 1.

**Figure 2.**
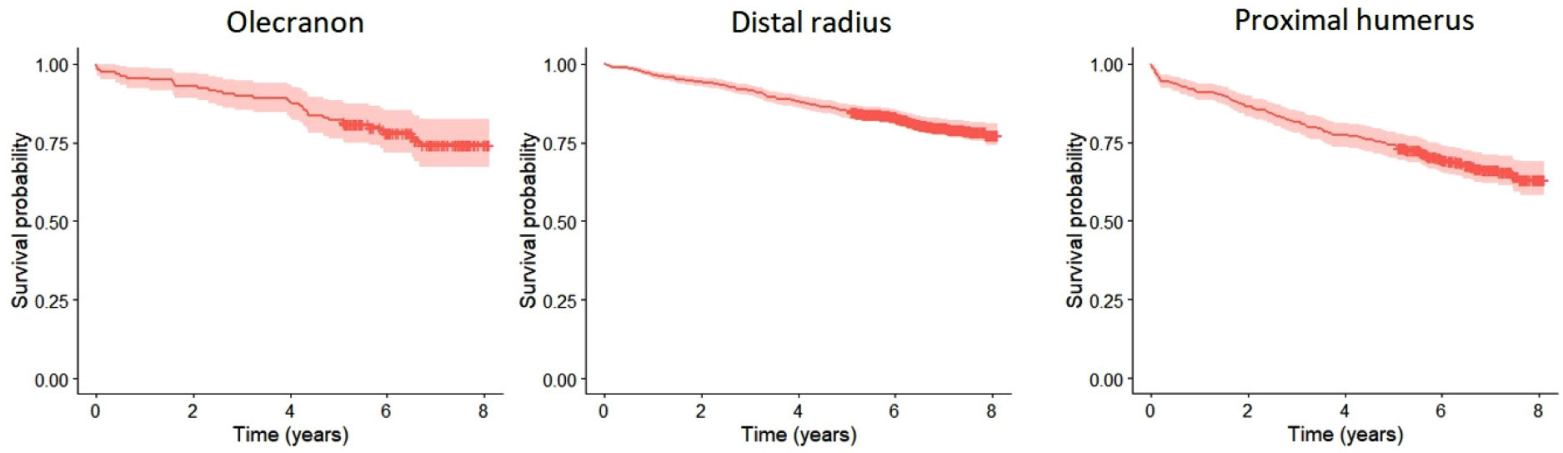
Kaplan-Meier analysis for mortality after olecranon, distal radius and proximal humerus fractures.

**Figure 3.**
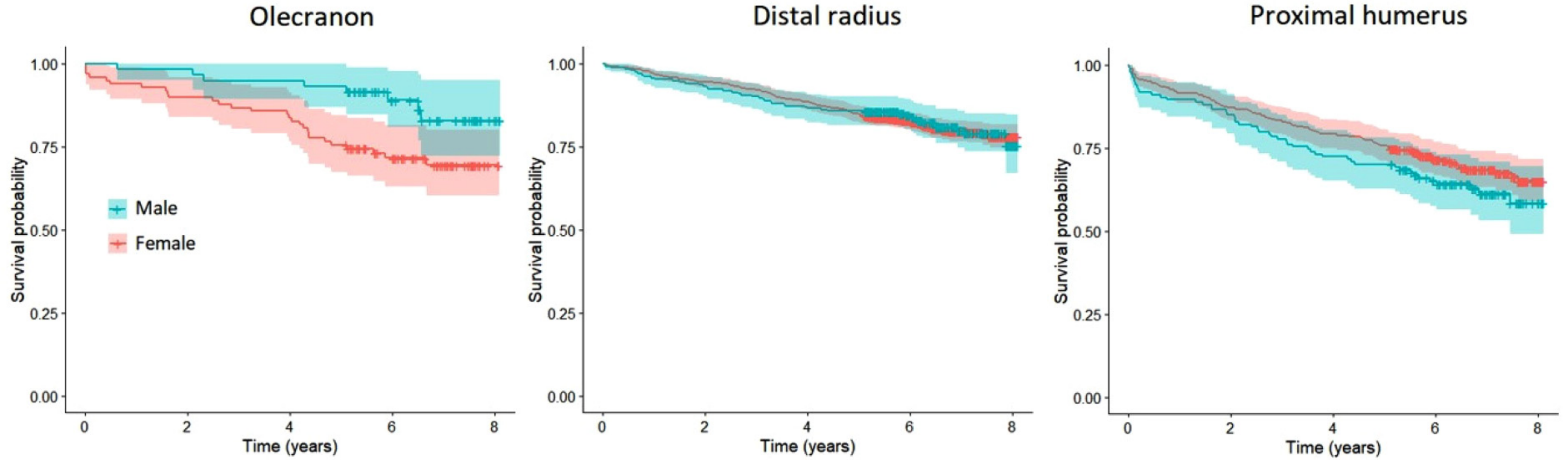
Sex-wise Kaplan-Meier analysis for mortality after olecranon, distal radius and proximal humerus fractures.

Mortality at 1 and 5 years for patients with an olecranon fracture was 4% and 13% (Figure 2,Table 2). There was no statistically significant difference in mortality between men and women when age was taken into consideration for patients with an olecranon fracture (HR 1.2, 95% CI 0.5–2.9) (Figure 3) based on the competing risk regression. For patients with a distal radius fracture mortality at 1 and 5 years was similar as in the olecranon fracture population, 3% and 13% (HR 1.3, 95% CI 0.79–2.15) but for patients with a proximal humerus fracture mortality was statistically significantly higher at both time points, 8% and 22% (HR 1.97, 95% CI 1.19–3.27) (Figure 2,Table 2). Unlike for patients with an olecranon fracture, male sex was associated with increased risk of mortality for patients with a distal radius or a proximal humerus fracture (HR 2.4, 95% CI 1.7–3.3; HR 2.4, 95% CI 1.7–3.5) (Figure 3).

**Table 2.** Risk of mortality after olecranon, distal radius and proximal humerus fractures in the competing risk analysis.

Fracture	Risk of mortality (% (95%CI))					
	1 year			5 years		
	M	F	ALL	M	F	ALL
**Olecranon**	1.7 (0.0–5.0)	5.1 (0.7–9.5)	3.8 (0.8–6.8)	6.8 (0.3–13.3)	16.3 (9.0–23.7)	12.7 (7.5–18.0)
**Distal radius**	4.3 (1.8–6.9)	3.0 (1.8–4.2)	3.3 (2.2–4.4)	12.7 (8.6–16.8)	13.6 (11.2–16.1)	13.4 (11.3–15.5)
**Proximal humerus**	9.0 (4.1–13.8)	7.9 (4.9–10.9)	8.2 (5.6–10.7)	26.1 (18.6–33.6)	20.8 (16.3–25.3)	22.3 (18.5–26.2)

CI: confidence interval; y: year; M: male; F: female.

Risk for a subsequent olecranon, distal radius, proximal humerus or hip fracture was 10% within 1 year and 14% within 5 years of follow up for patients with an olecranon fracture (Table 3). The risk of a subsequent fracture after a distal radius fracture was lower, 2% at 1 year and 5% within 5 years (HR 0.35, 95% CI 0.22–0.56), but for patients with a proximal humerus fracture the risks, 6% and 11%, did not differ statistically significantly from the risk after an olecranon fracture (HR 0.65, 95% CI 0.40–1.06).

**Table 3.** Risk of subsequent osteoporotic fractures (olecranon, distal radius, proximal humerus, hip) after an olecranon, distal radius, and proximal humerus fractures in the competing risk analysis.

Fracture	Risk of a subsequent fracture (% (95%CI))					
	1 year			5 years		
	M	F	ALL	M	F	ALL
Olecranon	6.8 (0.3–13.3)	12.2 (5.7–18.8)	10.2 (5.4–14.9)	8.5 (1.3–15.6)	17.3 (9.8–24.9)	14.0 (8.6–19.5)
Distal radius	3.6 (1.3–5.9)	1.9 (0.9–2.9)	2.3 (1.4–3.3)	4.0 (1.6–6.4)	5.6 (4.0–7.2)	5.2 (3.8–6.5)
Proximal humerus	6.0 (1.9–10.0)	6.0 (3.4–8.6)	6.0 (3.8–8.2)	9.0 (4.1–13.8)	11.7 (8.1–15.2)	10.9 (8.0–13.7)

CI: confidence interval; y: year; M: male; F:female.

## Discussion

In this study, we aimed to assess whether patients with olecranon fractures had comparable demographics, mortality, and risk for subsequent fractures compared to other common osteoporotic fractures in the upper extremity. The median age was similar to that of patients with a distal radius or a proximal humerus fracture. However, sex distribution was less female dominant and median age for male patients was lower than for patients with other upper extremity fractures. Risk of mortality was at the same level for patients with an olecranon or a distal radius fracture, but patients with a proximal humerus fracture had higher mortality rates. Patients with an olecranon fracture more often had subsequent fractures compared with patients with other upper extremity fractures.

In previous literature, the incidence of olecranon fractures starts to increase in both men and women at over 60 years of age and peaks at over 80 years.^15–17^ In our study, the median age of patients with olecranon fractures was 65 years, as was the median age of patients with distal radius fractures. However, the median age of men with olecranon fractures was significantly lower (51 years) than the median age of women with olecranon fractures (70 years). This is also seen in literature, where men with olecranon fractures are generally significantly younger than women.^16–18^ The age distribution was likewise uneven in all the analysed fracture groups, as could be expected based on previous studies, where women with upper extremity fractures were found to be generally older than men (62 years vs 46 years).^7^ In addition, a female predominance in patient population suffering a wrist fracture has been shown previously. In one study 88% of patients with wrist factures were women^6^ and in another study, the incidence for wrist fractures was approximately four times greater in women compared to men.^9^ In our data female predominance was seen in all analysed fracture groups.

Patients’ mortality after an olecranon fracture (1 year 4%, 5 years 13%) was comparable to that of patients with a distal radius fracture (1 year 3%, 5 years 13%; HR 1.3, 95% CI 0.79–2.15) in our material and mortality after a proximal humerus fracture was significantly higher (1 year 8%, 5 years 22%; HR 1.97, 95% CI 1.19–3.27). Interestingly, the reported 1 year mortality of Turku population aged between 50 and 74 years is below 1%.^19,20^ However, the generalizability of our results concerning the comparison of mortality after olecranon and distal radius fractures is questionable since the confidence interval includes 1.0. To our knowledge there is only one previous study assessing mortality solely after upper extremity fractures, in which patients with a distal radius fracture had lower mortality rates than patients with other upper extremity fractures, standardized mortality ratio (SMR) being 0.96. While assessing mortality of patients with proximal humerus fractures, it was considerably higher (SMR 2.0) compared to all the other fracture types. However, this study included only patients requiring inpatient care which probably has biased the studied population.^7^ In another study from Finland, where 1196 individuals aged 65 years were followed up for 12 years, proximal humerus fractures were related to excess mortality in men (HR 5.4), but not in women.^6^ A recent study analysing results of treatment of olecranon fractures in the elderly found 0.84 patient survival after olecranon fractures in a 1 year follow up.^21^ One study assessed mortality after hip, proximal humerus and wrist fractures, and found that the mortality was the lowest in patients with wrist fractures (1 year HR 1.2, 5 years HR 1.0) and intermediate high in patients with proximal humerus fractures (1 year HR 3.4, 5 years HR 1.9).^5^ Most likely these fractures do not have an effect on mortality as independent factors but having a certain upper extremity fracture might be associated with the overall health condition or fragility of a patient. We argue that if a patient is able to receive a fall on an extended upper extremity or with an elbow rather than just fall on their side and shoulder, their muscle strength, reflexes and overall health might be better. Overall health, on the other hand, naturally has an effect on survival.

In literature, it is shown that male sex is an independent risk factor for excess mortality concerning multiple fractures. Men had a HR of 2.0 for mortality compared to women after an upper extremity fracture requiring hospitalization^7^ and HR of 2.2 for mortality after any fracture in a population-based longitudinal study of elderly individuals.^6^ In the multivariate analysis, we found male sex to be an independent risk factor for mortality in distal radius and proximal humerus fracture groups.

The probability for a subsequent fracture after any fracture is known to be considerable regardless of fracture type or age. In a population-based study, the incidence of a subsequent fracture within 10 years after any fracture was 9–34% for men and 12–40% for women.^22^ A large cohort of elderly women with proximal humerus fractures was followed for 9.8 years and analysed for subsequent hip fractures. The first year HR for subsequent hip fracture was as high as 5.68, but the risk attenuated after the first year.^4^ To our knowledge, previously only one study has analysed the risk for a subsequent fractures after an olecranon fracture and they found a 4.1 risk ratio for a subsequent hip fracture after an olecranon fracture.^2^ We found a 10% probability of getting a subsequent fracture (distal radius, proximal humerus or hip) within the first year after an olecranon fracture and within 5 years the risk was 14%. The probabilities for a subsequent olecranon, distal radius or hip fracture after a proximal humerus fracture did not differ statistically from probabilities after an olecranon fracture (6% and 11%; HR 0.65, 95% CI 0.40–1.06), but after a distal radius fracture the risks for a subsequent olecranon, proximal humerus or hip fracture were statistically lower (2% and 5%; HR 0.35, 95% CI 0.22–0.56) than after an olecranon fracture. Concerning the comparison of the risk of subsequent fractures after an olecranon fracture and proximal humerus fracture, the confidence interval includes 1.0 which makes the generalizability of these results questionable. However, we consider these numbers comparable with the previous studies.^2,4^

Olecranon fractures are said to have osteoporotic features, such as low-bone mineral density association and a low-energy trauma as a predominant mechanism of injury.^23^ Proximal ulnar fractures have also been called fragility fractures for their predominant occurrence in elderly patients.^16^ The majority of the elderly patients suffering an olecranon fracture are women and in turn younger patients are men.^15–17,23^ Our findings strengthen this perception. As osteoporosis is more common in women than in men, olecranon fractures in elderly women indeed might have common features with other osteoporotic fractures of the upper extremity. Although we found that patients with olecranon fractures have osteoporotic features, the population of younger men with olecranon fractures still exists, and the fracture cannot be claimed to be solely osteoporotic. Consequently, when countering elderly women suffering an olecranon fracture, the prevention of future falls and prevention or treatment of possible osteoporosis should be kept in mind. Although, we would not yet recommend bone mineral density measurements or orthogeriatric evaluations routinely, as these are resource consuming means, instead, individual evaluation of the risks would be recommendable.

We acknowledge that our study has several limitations. Firstly, this was a retrospective diagnosis coding analysis with a limited number of variables. Secondly, all the data were gathered retrospectively from an electronic database and some information might be erroneously coded or missing as some fractures might have been miscoded. However, as these fractures are quite clearly defined, we do not think that this should have caused significant bias to our results. Thirdly, the coding did not include sidedness, and we therefore could not analyse whether there was a subsequent fracture to the same location or a same fracture on contralateral side. Fourthly, we could not count reliable enough incidence numbers, since it is possible that some patients had their fractures treated at the private sector clinics. However, in Finland the vast majority of trauma is treated in public sector, and we do not think that this has biased our data. On the other hand, the strength of our study is high number of systematically collected public sector consecutive patients in each fracture group. We also limited the search for inhabitants of city of Turku, Finland, to ensure as good coverage and follow-up as possible.

We conclude that patients with olecranon fractures have essentially similar demographic characteristics when compared to patients with distal radius fractures. Olecranon and distal radius fractures are associated with increased mortality and after a proximal humerus fracture the mortality is even higher. The risk of a subsequent osteoporotic fracture after an olecranon fracture was surprisingly high and statistically comparable to the risk after a proximal humerus fracture.

## References

[1] Eastell R Reid DM Compston J , et al. Secondary prevention of osteoporosis: when should a non-vertebral fracture be a trigger for action? QJM 2001; 94: 575–597. 11704688 10.1093/qjmed/94.11.575

[2] Lauritzen JB Lund B . Risk of hip fracture after osteoporosis fractures: 451 women with fracture of lumbar spine, olecranon, knee or ankle. Acta Orthop Scand 1993; 64: 297–300. 8322584 10.3109/17453679308993629

[3] Huntjens KMB Kosar S Van Geel TACM , et al. Risk of subsequent fracture and mortality within 5 years after a non-vertebral fracture. Osteoporos Int 2010; 21: 2075–2082. 20162259 10.1007/s00198-010-1178-5 PMC2974915

[4] Clinton J Mounce D Matsen FA , et al. Proximal humeral fracture as a risk factor for subsequent hip fractures. J Bone Joint Surg Am 2009; 91: 503–511. 19255209 10.2106/JBJS.G.01529 PMC2669747

[5] Shortt NL Robinson CM . Mortality after low-energy fractures in patients aged at least 45 years old. J Orthop Trauma 2005; 19: 396–400. 16003199 10.1097/01.bot.0000155311.04886.7e

[6] Piirtola M Vahlberg T Löppönen M , et al. Fractures as predictors of excess mortality in the aged - A population-based study with a 12-year follow-up. Eur J Epidemiol 2008; 23: 747–755. 18830674 10.1007/s10654-008-9289-4

[7] Somersalo A Paloneva J Kautiainen H , et al. Increased mortality after upper extremity fracture requiring inpatient care. Acta Orthop 2015; 86: 553–557. 10.3109/17453674.2015.1043833 PMC4564776 25909341

[8] Johnell O Kanis JA Odén A. , et al. Mortality after osteoporotic fractures. Osteoporos Int 2004; 15: 38–42. 14593451 10.1007/s00198-003-1490-4

[9] O’Neill TW Roy DK . How many people develop fractures with what outcome? Best Pract Res Clin Rheumatol 2005; 19: 879–895. 16301185 10.1016/j.berh.2005.06.003

[10] Kaplan EL Meier P . Nonparametric estimation from incomplete observations. J Am Stat Assoc 1958; 53: 457–481.

[11] Cox DR Regression models and life tables. J R Stat Soc Ser B 1972; 34: 187–220.

[12] Gray RJ . A class of KK-sample tests for comparing the cumulative incidence of a competing risk. Ann Stat 1988; 16: 1141–1154.

[13] R: A language and environment for statistical computing. R Foundation for Statistical Computing. *R Core Team* 2013.

[14] Therneau T (2022). A Package for Survival Analysis in R. R package version 3.40. https://CRAN.R-project.org/package=survival

[15] Court-Brown CM Caesar B . Epidemiology of adult fractures: a review. Injury 2006; 37: 691–697. 16814787 10.1016/j.injury.2006.04.130

[16] Duckworth AD Clement ND Aitken SA , et al. The epidemiology of fractures of the proximal ulna. Injury 2012; 43: 343–346. 22077988 10.1016/j.injury.2011.10.017

[17] Brüggemann A Mukka S Wolf O . Epidemiology, classification and treatment of olecranon fractures in adults: an observational study on 2462 fractures from the Swedish fracture register. Eur J Trauma Emerg Surg 2022; 48: 2255–2263. 34345928 10.1007/s00068-021-01765-2 PMC9192377

[18] Cantore M Candela V Sessa P , et al. Epidemiology of isolated olecranon fractures: a detailed survey on a large sample of patients in a suburban area. JSES Int 2022; 6: 309–314. 35252932 10.1016/j.jseint.2021.11.015 PMC8888171

[19] lounaistieto.fi. https://www.lounaistieto.fi/tilastot/vaesto/.

[20] Tilastokeskus. https://statfin.stat.fi/PxWeb/pxweb/fi/StatFin/StatFin__kuol/statfin_kuol_pxt_12ak.px/.

[21] Parkes J Limb R Quadiri S , et al. Complications and mortality associated with olecranon fractures in the elderly: a retrospective cohort comparison from a large level one trauma centre. Shoulder Elbow 2022; 14: 200–210. 35265187 10.1177/1758573221994860 PMC8899326

[22] Hansen L Petersen KD Eriksen SA , et al. Subsequent fracture rates in a nationwide population-based cohort study with a 10-year perspective. Osteoporos Int 2015; 26: 513–519. 25187120 10.1007/s00198-014-2875-2

[23] Park SC Gong HS Kim K , et al. Olecranon fractures have features of osteoporotic fracture. J Bone Metab 2017; 24: 175–181. 28955693 10.11005/jbm.2017.24.3.175 PMC5613022

